# Versatile microporous polymer-based supports for serial macromolecular crystallography

**DOI:** 10.1107/S2059798321007324

**Published:** 2021-08-23

**Authors:** Isabelle Martiel, John H. Beale, Agnieszka Karpik, Chia-Ying Huang, Laura Vera, Natacha Olieric, Maximilian Wranik, Ching-Ju Tsai, Jonas Mühle, Oskar Aurelius, Juliane John, Martin Högbom, Meitian Wang, May Marsh, Celestino Padeste

**Affiliations:** a Paul Scherrer Institute, Forschungsstrasse 111, 5232 Villigen, Switzerland; b Institute of Polymer Nanotechnology (INKA), FHNW University of Applied Sciences and Arts Northwestern Switzerland, 5210 Windisch, Switzerland; c Department of Biochemistry and Biophysics, Stockholm University, 106 91 Stockholm, Sweden; d MAX IV Laboratory, Lund University, Fotongatan 2, 224 84 Lund, Sweden

**Keywords:** fixed target, sample supports, SFX, serial crystallography

## Abstract

Serial data collection has emerged as a major tool for data collection at state-of-the-art light sources, such as microfocus beamlines at synchrotrons and X-ray free-electron lasers. Challenging targets, characterized by small crystal sizes, weak diffraction and stringent dose limits, benefit most from these methods. Here, the use of a thin support made of a polymer-based membrane for performing serial data collection or screening experiments is demonstrated. It is shown that these supports are suitable for a wide range of protein crystals suspended in liquids. The supports have also proved to be applicable to challenging cases such as membrane proteins growing in the sponge phase. The sample-deposition method is simple and robust, as well as flexible and adaptable to a variety of cases. It results in an optimally thin specimen providing low background while maintaining minute amounts of mother liquor around the crystals. The 2 × 2 mm area enables the deposition of up to several microlitres of liquid. Imaging and visualization of the crystals are straightforward on the highly transparent membrane. Thanks to their affordable fabrication, these supports have the potential to become an attractive option for serial experiments at synchrotrons and free-electron lasers.

## Abstract 2

Using a number of model and real-life practical cases, the use of sample supports made of a thin perforated cyclic olefin co­polymer membrane for serial crystallography that enable low-background serial data collection is described.

## 1. Introduction   

Serial crystallography currently encompasses two data-collection methods for modern structural biologists: single-shot, still exposures on thousands of microcrystals and small-rotation wedges from several to hundreds of microcrystals. The former can be performed at both X-ray free-electron laser (XFEL) and synchrotron light sources, whereas the latter can only be performed at a synchrotron. The management of the X-ray dose underpins the need for serial data-collection schemes at both sources (Bourenkov & Popov, 2010 ▸; Nass, 2019 ▸).

At XFELs, the high intensity of the X-ray pulses impedes the collection of multiple diffraction patterns at a given position, thus making serial femtosecond crystallography (SFX) the only possible data-collection method. At synchrotrons, serial rotation methods, inherited from virus crystallo­graphy (Fry *et al.*, 1999 ▸), have been in constant development. These developments, particularly high-flux microfocus beamlines (Evans *et al.*, 2011 ▸; Smith *et al.*, 2012 ▸; Rasmussen *et al.*, 2007 ▸), have enabled crystallography from ever smaller crystals with poorer diffraction. Serial rotation data collection at synchrotrons, now led by membrane-protein targets, consists of splitting the necessary dose over a number of small crystals (Cherezov *et al.*, 2009 ▸). Single-shot, still image data-collection methods at synchrotrons were driven by the XFEL SFX methods and spawned serial synchrotron crystallography (SSX; Stellato *et al.*, 2014 ▸; Botha *et al.*, 2015 ▸; Diederichs & Wang, 2017 ▸).

Other than the microfocus beamlines, the rise of serial crystallography at synchrotrons has been made possible by a number of recent developments. Firstly, state-of-the-art hardware and controls are now standard instrumentation at many synchrotrons, allowing rapid rastering procedures to find and center microcrystals even in optically opaque environments (Cherezov *et al.*, 2009 ▸; Zander *et al.*, 2015 ▸; Wojdyla *et al.*, 2016 ▸). Secondly, dedicated software tools are now widely available: *CrystFEL* and *cctbx* for SFX (Grosse-Kunstleve *et al.*, 2002 ▸; White *et al.*, 2016 ▸; Brewster *et al.*, 2018 ▸) and automatic data-set selection and merging pipelines at synchrotrons (Aller *et al.*, 2016 ▸; Guo *et al.*, 2018 ▸; Basu *et al.*, 2019 ▸). Finally, improved and novel sample-delivery methods have become available.

SFX and SSX initially relied upon purposely developed sample-injection methods (Schlichting, 2015 ▸). However, fixed-target setups quickly followed (Hunter *et al.*, 2014 ▸; Cohen *et al.*, 2014 ▸; Sherrell *et al.*, 2015 ▸; Roedig *et al.*, 2015 ▸; Zarrine-Afsar *et al.*, 2012 ▸; Coquelle *et al.*, 2015 ▸; Lyubimov *et al.*, 2015 ▸), with data-collection strategies broadly based upon rapid rastering strategies (Martiel *et al.*, 2019 ▸; Cheng, 2020 ▸; Schlichting, 2015 ▸). These methods therefore rely upon fast scanning stages with the protein crystal ‘fixed’ by a variety of different supports.

Supports for fixed-target crystallography, often called chips, can be classified into two broad groups based on the chip material: either silicon or polymer. Silicon-based supports are directly fabricated by lithography and/or etching techniques and have windows at defined positions where loaded crystals are sequentially exposed to the X-ray beam. These windows are meant to act as sites for deposited crystals and avoid the strong reflections of the silicon (111) crystal plane. Initially, chips were composed of a layered silicon/silicon nitride composite with wells chemically etched into the silicon up to a 150 nm silicon nitride base (Murray *et al.*, 2015 ▸; Huang *et al.*, 2016 ▸; Hunter *et al.*, 2014 ▸). The wells are watertight and can also be used as enclosures on both sides of the sample (Opara *et al.*, 2017 ▸).

However, the most established systems feature a silicon chip with regularly spaced apertures of a size matched to the crystal or beam (Oghbaey *et al.*, 2016 ▸; Lieske *et al.*, 2019 ▸; Mehrabi *et al.*, 2020 ▸; Roedig *et al.*, 2016 ▸; Zander *et al.*, 2015 ▸). Different geometries exist for the apertures, either as holes with straight walls (Roedig *et al.*, 2015 ▸) or pyramidal cavities (Mueller *et al.*, 2015 ▸), and their precise placement allows rapid alignment strategies (Sherrell *et al.*, 2015 ▸). To facilitate loading, the silicon chips are rendered hydrophilic by glow-discharging the surface before deposition (Ebrahim *et al.*, 2019 ▸). After deposition of microcrystal solution, the mother liquor is removed through the apertures by blotting or by applying a vacuum. For room-temperature use, the chips are often enclosed between polymer films and/or kept in a humid environment. Silicon manufacturing imposes some geometrical constraints and is costly, but the chips can often be washed and reused in spite of their fragility. The advantage of their low X-ray absorption properties becomes especially evident when the mother liquor is removed.

The second group of supports comprises a large variety of polymeric matrices. Polymers offer large manufacturing freedom and affordable costs, but their pronounced X-ray scattering properties necessitate structures that are as thin as possible. Commercial nylon or polyimide loops (Gati *et al.*, 2014 ▸) and micromeshes (Cohen *et al.*, 2014 ▸) have been used for cryogenic serial crystallography at synchrotrons. Their overall dimensions are typically below 1 mm and they have large openings where crystals can be suspended in mother liquor. A further developed version of the polyimide micromesh provides smaller openings for maintaining small microcrystals (Guo *et al.*, 2018 ▸). Several multi-crystal mounts made out of laser-cut polycarbonate have also been designed specifically for serial crystallography. Baxter *et al.* (2016 ▸) developed a multiport mount for use at either room temperature with film enclosures or in cryogenic conditions. Barnes *et al.* (2019 ▸) designed a square mount in a cryogenic format that is compatible with UV imaging for crystal pre­location. Carbon films deposited on polymeric grids have been used to deliver 2D crystals (Feld *et al.*, 2015 ▸). Off-the-shelf nylon meshes have also been used as scaffolds for holding microcrystals between polymer films (Lee *et al.*, 2019 ▸; Park *et al.*, 2020 ▸). Microcrystal slurries (Doak *et al.*, 2018 ▸) and viscous mixtures (Lee *et al.*, 2020 ▸) have even been simply sandwiched between polymer films or multilayer graphene films (Shelby *et al.*, 2020 ▸). These methods are cost-effective, but the substantial amounts of retained mother liquor or other surrounding media increases the X-ray scattering background. Blotting of the mother liquor for reduced scattering requires micro-sized pores to retain microcrystals (Feiler *et al.*, 2019 ▸; Zander *et al.*, 2016 ▸; Guo *et al.*, 2018 ▸), such as the 5 µm wide pores drilled in the 21 µm thick polyimide membrane by the XtalTool system (Feiler *et al.*, 2019 ▸). However, how to best control the amount of liquid removed or retained on the chip is a generally unexplored question.

Here, we present a novel polymer-based support and an associated deposition method, with the aim of providing ultralow-background conditions for serial data collection. The supports themselves consist of a thin 2–3 µm thick cyclic olefin co­polymer (COC) membrane perforated with a dense array of 2–3 µm sized holes and suspended on a rigid frame. Compared with other polymer-based supports, the freestanding area of the membrane is large, typically 2 × 2 mm, and stable without any thickening or reinforcement. The fabrication method (Karpik *et al.*, 2020 ▸) combines the precision of microfabrication with the versatility of polymer processing and affordable fabrication cost thanks to wafer-scale batch production.

The deposition method was carefully studied using a variety of cases, including the lipid sponge phase, which is notoriously difficult to extract crystals from. The method was optimized by taking advantage of the membrane surface properties. Ultimately, this involved retaining only the minimum amount of embedding liquid around the crystals, thus minimizing the X-ray scattering background, while still preserving the diffraction quality of the crystals. The system proved to be highly adaptable and amenable to a variety of cases, including membrane proteins grown in vapor-diffusion drops or in sponge phase. This preparation method can be useful at many stages of a project; for example, when searching for highly diffracting crystals from drops full of microcrystals. This paper reports a selection of data sets collected on the synchrotron microfocus beamlines of the Swiss Light Source (SLS) using the polymer-based supports.

## 2. Materials and methods   

### 2.1. Fabrication of the supports   

Chip fabrication was performed as described in Karpik *et al.* (2020 ▸). Briefly, an imprint stamp with a dense array of pillars of 5–6 µm in height was produced from a 10 cm silicon wafer by photolithography and reactive ion etching. A double layer of spin-coated polymer [300 nm water-soluble polyvinyl­pyrrolidone (PVP) and 3 µm COC] on a silicon substate was then thermally imprinted with the pillar array. Etching was used to remove the residual layer inside the imprinted holes, before polylactic acid frames were directly 3D-printed onto the membrane using a fused deposition modeling printer (Original Prusa i3 MK3, Prusa Research, Prague). The individual chips were then cut away from each other and released from the wafer by dissolving the PVP bottom layer in water. Finally, in order to equilibrate the surface properties, the individual chips were dried in air and kept for at least ten days under ambient conditions before use.

The nanoimprint-based fabrication of the central membrane allows some design flexibility of the imprinted micropattern. For instance, the sizes, densities and arrangements of the perforations can be adapted for specific needs by the design of the imprint stamp. Furthermore, the thickness of the membrane is determined by the spin-coating parameters that are used. Throughout this study, we used membranes of 3 µm in thickness with square symmetric arrangements of 2 µm wide perforations with a periodicity of 4 µm. As an additional feature, some of the membranes contained a superlattice achieved by leaving out every 25th row of holes, resulting in a 100 × 100 µm array that can be used as an internal scale.

### 2.2. Protein production and crystallization   

#### 2.2.1. Preparation of lysozyme microcrystals   

Hen egg-white lysozyme (HEWL), procured from Sigma–Aldrich GmbH, Buchs, Switzerland (catalogue No. L2879), was dissolved in 0.1 *M* sodium actetate pH 3.0 to a final concentration of 25 mg ml^−1^. 0.5 ml of the HEWL solution was mixed in a 1:1 ratio with 21%(*w*/*v*) NaCl, 8%(*w*/*v*) polyethylene glycol (PEG) 6000, 0.1 *M* sodium acetate pH 3.0 in a centrifuge tube at 20°C (based on Weinert *et al.*, 2017 ▸). The tube was inverted ten times and left overnight on a revolver/rotator (Thermo Fisher Scientific) at 20°C. The crystal size (the mean longest dimension) and concentration were determined using a Bright–Line counting chamber (Thermo Fisher Scientific) and were approximately 25 µm and 3.4 × 10^6^, respectively.

#### 2.2.2. Preparation of thaumatin microcrystals   

Thaumatin from *Thaumatococcus danielii* (catalogue No. T7638) was purchased from Sigma–Aldrich GmbH, Buchs, Switzerland and dissolved in double-distilled H_2_O to a final concentration of 50 mg ml^−1^. 0.5 ml of the thaumatin solution was mixed in a 1:1 ratio with 40%(*w*/*v*) sodium potassium tartrate, 0.1 *M* bis-Tris propane pH 6.5 in a centrifuge tube at 20°C (based on Nass *et al.*, 2020 ▸). The centrifuge tube was then briefly vortexed and left overnight on a revolver/rotator at 20°C. The mean crystal size and concentration were 10 µm and 1.9 × 10^7^, respectively.

#### 2.2.3. Preparation of insulin microcrystals   

Insulin from porcine pancreas (catalogue No. I5523) was purchased from Sigma–Aldrich GmbH, Buchs, Switzerland and dissolved in 50 m*M* Na_2_HPO_4_, 10 m*M* EDTA pH 10.5 to a final concentration of 25 mg ml^−1^. Crystals were grown using two different methods for different parts of the investigation. The crystals used in Sections 3.1 and 3.4 were grown in batch by mixing 0.5 ml of the insulin solution in a 1:1 ratio with the crystallization buffer [25%(*w*/*v*) PEG 6000, 0.1 *M* bis-Tris propane pH 7.5, 0.2 *M* sodium bromide] in a centrifuge tube at 20°C. The centrifuge tube was briefly vortexed and left on a revolver/rotator at 20°C. The mean crystal size and concentration were 30 µm and 1.5 × 10^6^, respectively. The crystals used in Sections 3.2 and 3.3 were grown overnight at 20°C in 24-well sitting-drop vapor-diffusion CrysChem plates (Hampton Research, USA) against 500 µl reservoir. The drops contained 2 µl insulin solution and 2 µl crystallization buffer.

#### 2.2.4. Preparation of tubulin–DARPin D1 complex (TD1) crystals   

DARPin D1 was prepared as described previously by Pecqueur *et al.* (2012 ▸). Tubulin from bovine brain was purchased from the Centro de Investigaciones Biológicas (Microtubule Stabilizing Agents Group), CSIC, Madrid, Spain. The tubulin–DARPin D1 (TD1) complex was formed by mixing the respective components in a 1:1.1 molar ratio. The TD1 complex was batch-crystallized overnight at 20°C in 0.6 ml centrifuge tubes. 10 µl TD1 complex solution at 18.9 mg ml^−1^ was mixed with 10 µl precipitant solution consisting of 26%(*w*/*v*) PEG 3000, 0.2 *M* ammonium sulfate, 0.1 *M* bis-Tris methane pH 5.5, and 2 µl seed solution was added in order to ensure controlled homogenous nucleation. The seed solution was made of spun-down (a few seconds at 1200 rev min^−1^) concentrated crystalline material obtained by the hanging-drop vapor-diffusion method [drop size 2 µl, drop ratio 1:1, 18%(*w*/*v*) PEG 3000]. The average size of the final crystals (monoclinic needles) was about 120 × 10 × 5 µm.

#### 2.2.5. Preparation of ribonucleotide reductase radical-generating subunit (EcR2a) crystals   

The construct preparation, protein expression, purification and metal loading of class Ia ribonucleotide reductase radical-generating subunit from *Escherichia coli* (EcR2a) were performed as described in Aurelius *et al.* (manuscript in preparation). Crystals were grown in 24-well sitting-drop plates by vapor diffusion. EcR2a at 21 mg ml^−1^ was centrifuged at 14 000*g* for 10 min at 4°C prior to crystallization. 2–3 µl protein solution was mixed with 1 µl reservoir solution in a 24-well sitting-drop plate with a 300 µl reservoir. The reservoir conditions were 37%(*w*/*v*) PAA 2100, 100 m*M* HEPES 7.0, 400 m*M* NaCl, 200 m*M* ammonium sulfate or 32%(*w*/*v*) PAA 2100, 100 m*M* HEPES 7.0, 150 m*M* NaCl, 100 m*M* malonate. Hexagonal-shaped crystals were visible in most drops after 12 h incubation at 21°C.

#### 2.2.6. Preparation of rhodopsin–miniG_o_ complex crystals   

Crystals were prepared essentially as described in Tsai *et al.* (2018 ▸), but the crystallization was performed in hanging-drop vapor-diffusion EasyXtal 15-well plates (Qiagen) with 0.5 ml crystallization buffer in the reservoir. Crystallization drops were set up at 4°C by mixing 1.5–3 µl protein solution with an equal volume of crystallization buffer consisting of 0.1 *M* MES pH 5.5, 10–25%(*w*/*v*) PEG 4000.

#### 2.2.7. Preparation of peptide transporter (PepT_St_) crystals in sponge phase   

PepT_St_ from *Streptococcus thermophilus* was produced recombinantly in *E. coli* and purified as described in Lyons *et al.* (2014 ▸). The *in meso* crystallization trials were performed by following the established protocol using two 100 µl Hamilton glass syringes and a coupler (Caffrey & Cherezov, 2009 ▸). The PepT_St_-laden LCP was obtained by mixing 10 mg ml^−1^ protein solution with 1-(7*Z*-pentadecen­oyl)-rac-glycerol (7.8 MAG) in a 1:1 volume ratio. 20 µl of the LCP PepT_St_–MAG mixture was injected into a 100 µl Hamilton glass syringe containing 70 µl screen solution. The screen solution used for PepT_St_ was 325 m*M* NH_4_H_2_PO_4_, 100 m*M* HEPES pH 7.0, 21–22%(*v*/*v*) PEG 400, 10 m*M* alanine–phenylalanine dipeptide. The crystallization was performed at 20°C and crystals were harvested after seven days. The LCP turned into sponge phase during crystallization.

### 2.3. Deposition on chips   

**Figure 1:**
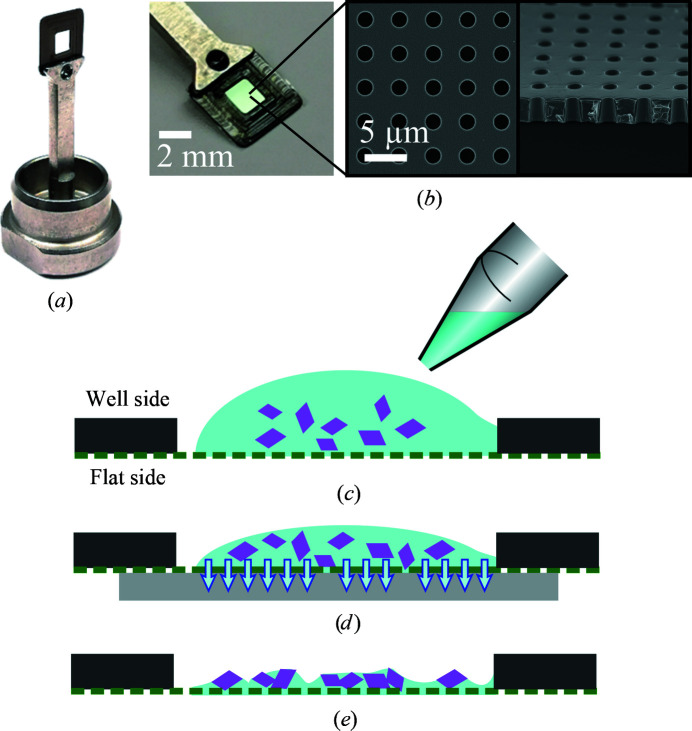
(*a*) Chip mounted on a SPINE pin-like steel holder. (*b*) Close-up view of a chip glued on a holder, with SEM images of the microporous membrane in the inset: a top view showing the hole dimension and spacing (left) and a side view of the flipped and cut-off membrane showing its thickness (right). Both inset images are on the same scale. (*c*, *d*, *e*) Chip-loading principle. (*c*) A liquid suspension of crystals is deposited on the well side of the chip. (*d*) The excess liquid is blotted away by applying a flat piece of blotting paper on the flat side. (*e*) Only a minimal amount of liquid is left. The sample is then immediately preserved from dehydration by flash-cooling in liquid nitrogen or cool nitrogen gas. The schemes are not to scale.

The chips were fixed to metal bases with 1–2 µl of cyano­acrylate glue (Cementit CA 10, Merz + Benteli AG) deposited in the dedicated hole (Figs. 1 ▸ *a* and 1 ▸ *b*). The glue is soluble in acetone and therefore allows recycling of the metal bases by placing the pins in an acetone bath. In the case of sponge-phase-grown PepT_St_ crystals, the chips were pretreated with a drop of silanizing agent (MiTeGen, IMISX beta kit) to render them hydrophobic and were then washed with water.

Prior to chip deposition, some of the soluble protein crystals were mixed with a deposition solution (see Table 1 ▸). For the batch-grown crystals (lysozyme and thaumatin), 1 µl of the sedimented crystal pellet was mixed with 50 µl of the deposition solution in a centrifuge tube. For crystals grown in sitting drops (insulin and EcR2a), 0.2 µl of the crystallization drop was mixed with 0.8 µl deposition solution. Once mixed, 2 µl of the crystal-deposition solution mixture was used for chip loading.

Rhodopsin–miniG_o_ crystals were directly deposited from the hanging drop. 1 µl of mixture (or drop for rhodopsin–miniG_o_) was deposited onto the well side of the chip (Supplementary Fig. S1*a* ).

For the sponge-phase-grown PepT_St_ crystals, about 2 µl of biphasic lipid phase–precipitant sample was deposited directly from the syringe.

Chips were blotted on the flat side by applying a stripe of thin chromatography paper (grade 1 CHR, Whatman) flat over the full surface of the membrane. The solution was blotted away by applying gentle pressure with a finger on the back of the paper, to ensure a good contact between the paper and the chip, whilst visually monitoring the surface of the drop receding through the membrane as blotting progressed. This process can be performed either under visual control by eye (Supplementary Figs. S1*a* and S1*c* ) or using a microscope (Supplementary Figs. S1*d* and S1*e* ). The goal of blotting is to remove as much of the crystallization solution as necessary without over-drying the crystals (see Supplementary Fig. S2). As soon as the liquid had been blotted away, the chips were immediately flash-cooled either in the on-axis beamline cryojet or, alternatively, by plunge-cooling into liquid nitrogen. Liquid removal during blotting is not only essential to reduce the ultimate background but also helps to prevent chip cracking, although this phenomenon was largely mitigated by successive iterations of chip-design improvement (Karpik *et al.*, 2020 ▸). When using the plunge-freezing method, a UniPuck containing 16 samples could be filled in about 10 min. All of the samples prepared in this work were blotted under visual control by eye.

### 2.4. Data collection and data processing   

**Figure 2:**
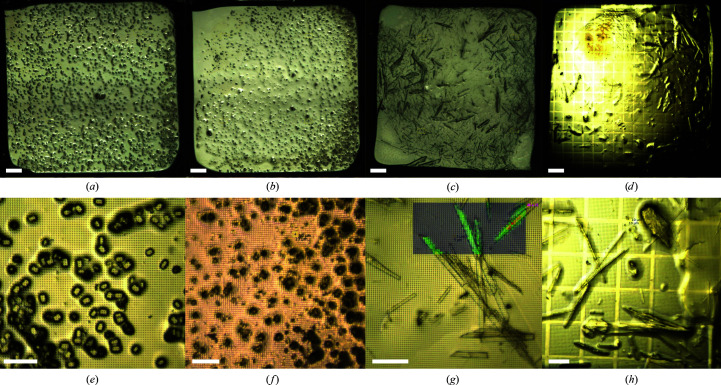
Online microscope views of chips prepared with some of the tested protein crystals at two magnifications: (*a*, *e*) hen-egg white lysozyme microcrystals, (*b*, *f*) thaumatin microcrystals, (*c*, *g*) TD1 crystals and (*d*, *h*) rhodopsin–miniG_o_ crystals. Scale bars are 200 µm in the top panels and 50 µm in the bottom panel. (*g*) shows the grid-scan evaluation results in color, illustrating diffraction from the crystals. (*a*)–(*c*) are stitched images of intermediate magnification suitable for the detection of crystals for prelocation purposes.

Chips were either mounted automatically with the TELL sample changer (Martiel *et al.*, 2020 ▸) or manually mounted by direct flash-cooling in the cryojet. Fig. 2 ▸ shows some images of cryocooled samples taken with a beamline on-axis microscope.

#### 2.4.1. Data collection and handling for comparative analyses   

Data for each protein and each cryoprotectant in Section 3.1 were collected from ten random crystals on the chip surface on SLS beamline PX1. A 60° wedge (0.1° oscillation, 0.01 s exposure) was collected from each crystal at 12.4 keV. The beam size and attenuation were tailored to the protein crystal: 30 µm and 0.2 transmission for insulin, 10 µm and 0.3 transmission for thaumatin and 25 µm and 0.2 transmission for HEWL. For comparison against similar crystals in loops and micro-meshes (MiTeGen; Section 3.4), data were collected in a similar manner but from single crystals mounted in loops or multiple crystals in micro-meshes, as required. Ten data sets were from each of these could then be compared with the chip-mounted crystals.

All of the data were reduced and scaled using *DIALS* (Winter *et al.*, 2018 ▸). Data were cut at a CC_1/2_ of 0.3 and the statistics were collected for further analysis. One-way ANOVA followed by Dunnett’s multiple comparisons tests were performed using *Prism* 9 (GraphPad).

#### 2.4.2. Data collection and handling for structure solution   

Data sets were collected in two different ways: rotations and stills. Rotation-wedge data sets were collected and processed using *XDS* and *XSCALE* (Kabsch, 2010 ▸) using the automatic serial crystallography pipeline of the macromolecular crystallography (MX) beamlines at the SLS (Basu *et al.*, 2019 ▸). Stills data sets were collected using the grid-scan tool (Wojdyla *et al.*, 2016 ▸) with a grid covering the whole chip, and were processed with the *CrystFEL* suite version 0.7 or 0.8 (White *et al.*, 2016 ▸; see Table 2 ▸ for the specific data-collection parameters for each data set). All phases were solved by molecular replacement and the structure refinements were carried out using the *Phenix* suite (Liebschner *et al.*, 2019 ▸). The search models used were PDB entry 5ne0 for lysozyme, PDB entry 4axr for thaumatin, PDB entry 5d53 for insulin, PDB entry 5nqu for TD1, PDB entry 1mxr for EcR2a and PDB entry 4xnj for PepT_St_. For background studies, single images were recorded with 100 ms exposure and 0.5° rotation at a 130 mm detector distance at 12.397 keV with a 10 × 10 µm beam and full transmission (3.9 × 10^11^ photons s^−1^). The collimator was placed 4 mm before the sample and the beamstop was placed 25 mm behind it, which are standard positions for data collection at the SLS. Powder plots were calculated using *ALBULA* (Dectris, Switzerland). Mean diffraction-weighted dose calculations for the rotation and still data sets were performed using *RADDOSE*-3*D* (Bury *et al.*, 2018 ▸).

## 3. Results   

### 3.1. Initial optimization of crystal deposition on the chip   

**Figure 3:**
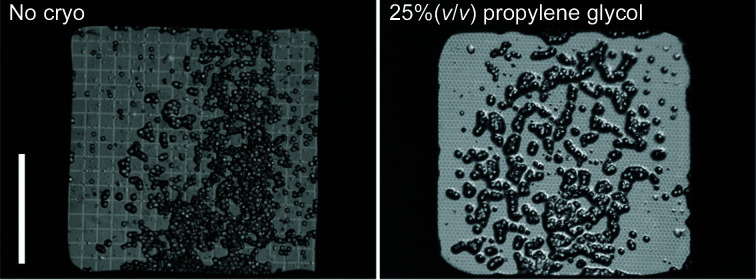
Visual comparison between chips loaded with insulin crystals from solutions containing no cryoprotectant (left panel) and 25%(*v*/*v*) propylene glycol (right panel). The scale bar (left panel) denotes 1 mm.

Our initial chip-deposition tests with water-soluble proteins showed that direct deposition of a microcrystal slurry without any cryoprotection, followed by blotting and flash-cooling, was not satisfactory. Fig. 3 ▸ illustrates the positive impact of the addition of a cryoprotectant on the loading of insulin crystals onto the chips. The number of insulin crystals that visually appear to be surrounded by solution is markedly higher in the presence of a cryoprotectant. Crystal slurries without cryoprotectant also often displayed irregular distributions of clumped crystals, large blobs of mother liquor (a source of ice rings) or completely dry sections of the chip (Supplementary Figs. S3*a* and S3*b* ). Although it was possible to collect data from some protein crystals without cryoprotectant (see Section 3.2), the addition of cryoprotectant improved data quality in terms of the resolution of Bragg reflections. The addition of cryoprotectants, such as propylene glycol or PEG 400, therefore yielded better results in terms of chip loading and data collection. A more thorough analysis of the impact of cryoprotectants is decribed in Section 3.4.

### 3.2. Data collection for structure solution   

To assess the quality of structures that can be obtained from chip-mounted crystals, diffraction data were collected from a range of proteins. These proteins included the insulin, thaumatin and HEWL that were used in initial tests, and also TD1, a tubulin–DARPin D1 complex, EcR2a, a class Ia ribo­nucleotide reductase radical-generating subunit, rhodopsin–miniG_o_ and PepT_st_, a bacterial peptide transporter. TD1 and EcR2a are both soluble proteins with crystals grown using vapor diffusion, rhodopsin–miniG_o_ is a membrane protein with crystals grown in vapor-diffusion plates and PepT_st_ is a membrane protein where crystals are obtained using LCP methods.

#### 3.2.1. Data-collection results   

An analysis of the crystal orientation (Supplementary Fig. S4) confirms that, as might be expected for a flat support, the crystals are prone to preferential orientations once deposited on the chip. Nevertheless, for the samples used in this study, the observed degree of preferential orientation was not an obstacle to the collection of complete data sets from a single chip, even by grid scan without tilt or rotation (Table 2 ▸). This was also the case for the needle-like *P*2_1_ TD1 crystals, although the completeness remained slightly lower (94%) than for the other cases.

**Figure 4:**
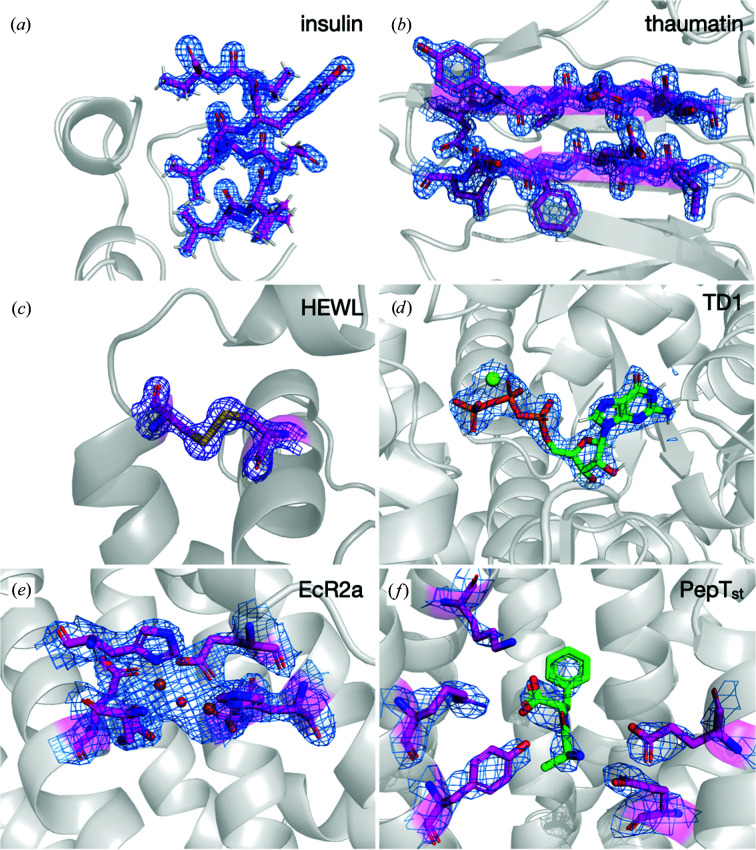
Images of the parts of the protein structure and density for the solved protein structures. (*a*) Insulin (σ = 2.0), (*b*) thaumatin (σ = 2.0), (*c*) HEWL (σ = 2.0), (*d*) TD1 (σ = 2.0), (*e*) EcR2a (σ = 1.0) and (*f*) PepT_st_ (σ = 1.0).

Samples of the soluble proteins insulin, thaumatin and HEWL (Figs. 4 ▸ *a*, 4 ▸ *b* and 4 ▸ *c*) readily yielded high-quality and high-resolution data sets (Supplementary Fig. S5) by the collection of rotation wedges from a small fraction of the hundreds to thousands of microcrystals deposited on the chip using the serial data-collection pipeline CY+ (Basu *et al.*, 2019 ▸). For the soluble proteins TD1 and EcR2a (Figs. 4 ▸ *d* and 4 ▸ *e*), data collection was performed by both rotation wedges and still grid scans on two different chips prepared from the same sample.

The quality and resolution of the PepT_st_ data set (Fig. 4 ▸ *f*, Table 2 ▸), obtained from sponge-phase-grown crystals, were also moderate in comparison to reported structures (Huang *et al.*, 2016 ▸, 2018 ▸). This was related to the low number of crystals and the still imperfect deposition method that was used. However, successful blotting of sponge phase was demonstrated, offering new possibilities for handling and background reduction for sponge-phase samples.

### 3.3. Background scattering characterization   

**Figure 5:**
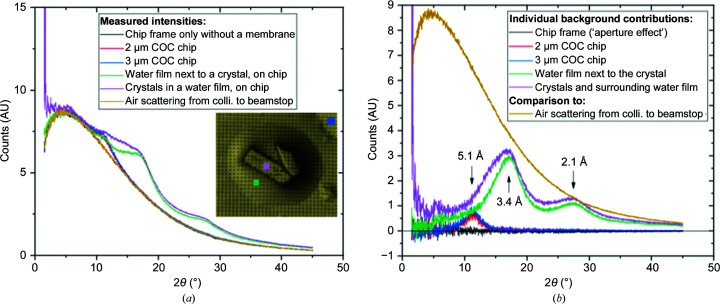
(*a*) Total background measurements and (*b*) separated contributions from the different sample components. The curves in (*b*) were obtained by subtraction of the air scattering from the relevant curves. The inset microscope picture in (*a*) shows the position of the beam on the sample for the most intense background curves as squares of the same color as the respective curve. The colored squares are 10 µm in size, representing the beam size.

The chips were specifically designed to reduce the mounting material in the X-ray path to an absolute minimum (2–3 µm), and the loading method was optimized to reduce the excess of surrounding mother liquor. An analysis of the background scatter from the chips was therefore conducted to acertain its success. Fig. 5 ▸ shows the background curves measured on an optimally deposited sample on a chip. The inset in Fig. 5 ▸(*a*) shows an approximately 10 µm thick plate-like crystal (as observed from the crystal morphology in the drop before deposition) surrounded with a liquid meniscus. Background curves were measured at different places on the chip (Fig. 5 ▸ *a*, inset). The basal air scattering was then subtracted to obtain the curves corresponding to the individual contributions of the different components of the sample, solution and mount (Fig. 5 ▸ *b*).

The background contribution from the chips themselves (blue and red curves for the 3 and 2 µm thick membranes, respectively) is by far the lowest of the background contributions. Its only maximum is situated at 5.1 Å resolution, and therefore does not interfere with weaker high-resolution reflections. The broadness of this peak is due to the amorphous state of the polymer film.

Background curves were measured at the crystal position (purple curve) and on the meniscus nearby (green curve). As typically observed for the background from water-based media, the maximum background level is at around 3.4 Å resolution, with a secondary maximum at 2.1 Å resolution. The background signal measured at the position of the crystal (purple curve) was found to be similar to that measured next to it in the liquid meniscus (green curve), suggesting that their thicknesses are comparable. This shows that the blotting was effective and almost no extra liquid is present on top of the crystal. In other words, at the crystal position the X-ray beam crosses the crystal on its very thin support, with essentially no mother liquor on top, which is an optimal situation for background reduction. At the same time, the crystal remains surrounded by a visible meniscus of liquid for optimal preservation.

The background caused by air scattering along the direct beam path, carefully reduced at MX beamlines by a collimator a few millimetres before the sample and a beamstop placed a couple of centimetres behind, is in practice generally considered to be a negligible source of background in crystallo­graphy experiments at MX synchrotron beamlines, including serial experiments with challenging targets. The background contribution from the crystal and chip (purple curve) was found to be lower than the beamline-related air-scattering background (yellow curve).

As expected from the dimensions of the liquid and polymer layers, the background signal from crystals deposited on the chips was found to be significantly lower than the background obtained from the same crystals harvested on a polyimide micromesh (Supplementary Fig. S6), where both the plastic support and the retained liquid can be thicker. This advantage is particularly striking in the region of the water ring, which is the resolution range that is most relevant for many challenging projets, where background reduction may directly result in resolution improvement. In this water-ring resolution range, the background contribution from the precipitant retained on the mesh was found to largely exceed that of air along the direct beam path, while the background levels in the chip-deposed samples remained at a fraction of the air scattering.

### 3.4. Correlating wetting angle and diffraction for soluble protein buffers   

**Figure 6:**
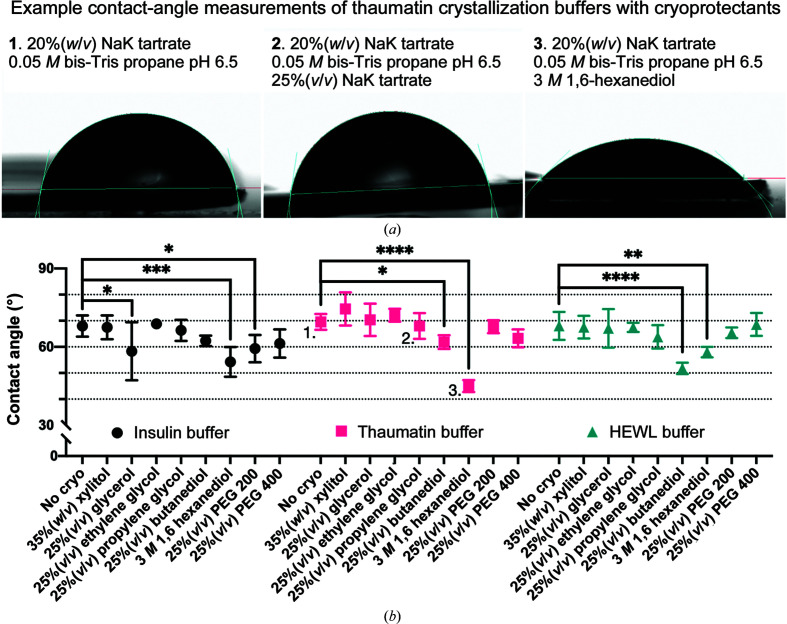
An analysis of how the addition of different common cryoprotectant compounds to a series of crystallization solutions changes their chip–solution contact angle. (*a*) Example images of contact-angle measurements for a thaumatin crystallization buffer without (1) and with (2 and 3) different cryoprotectants. (*b*) A comparison of the effects that different cryoprotectants have on the chip–solution contact angle of three different protein crystallization buffers (see Table 3 ▸). The measurements corresponding to the images in (*a*) have been labeled. The mean and 95% confidence limits of each measurement have been plotted. The asterisks indicate the significance of the *p*-value obtained from a one-way ANOVA comparison between the ‘no cryo’ buffer and the buffer plus an additive (*, <0.0332; **, <0.0021; ***, <0.0002; ****, <0.0001).

Wetting is the term used to the describe the behavior of a solution when that solution comes into contact with a solid surface in an gaseous environment. At standard temperature and pressure (20°C, 101.325 kPa), the interaction will be primarily governed by the cohesive and adhesive forces within the liquid and between the liquid and surface, respectively, as well as the surface roughness (Kontogeorgis & Kiil, 2016 ▸). Wetting can be characterized by the contact angle of the liquid on the material surface (Fig. 6 ▸ *a*). A strong liquid–surface force and a weak liquid cohesive force will flatten droplets as they come into contact with the surface (Fig. 6 ▸ *a*, right panel), whereas the reverse will result in droplets that ball up (Fig. 6 ▸ *a*, left panel). Measuring the contact angle between the surface and droplet edge allows the quantification of the result of these forces. Our hypothesis was that finding buffer and cryoprotectant mixtures that reduce the contact angle would improve the loading and ultimately the data quality.

To test the correlation between the chip–solution contact angle and the diffraction quality of the resulting diffraction data, a series of cryoprotectants (McFerrin & Snell, 2002 ▸; Garman & Owen, 2006 ▸) were selected for investigation (Table 3 ▸). Insulin, thaumatin and HEWL were selected as test proteins, partly due to their availability and reliability but also because they can be crystallized in a range of different buffers (Table 3 ▸). These buffers very broadly represent the types of solutions used to crystallize proteins: mainly PEG, mainly salt, and PEG and salt.

The protein crystallization buffer was mixed with each cryoprotectant at a typical concentration, and the contact angle between the drop and the chip surface was measured (Fig. 6 ▸ *b*). For each of the insulin, thaumatin and HEWL crystallization buffers, no statistically significant difference in the contact angle was observed upon the addition of the cryoprotectants xylitol, ethylene glycol, propylene glycol and PEG 400. However, the addition of 1,6-hexanediol significantly altered the contact angle of every buffer. The addition of glycerol and PEG 200 were only significant in the case of the insulin buffer, whereas butanediol significantly altered the thaumatin and HEWL buffers.

To correlate these results with diffraction data, microcrystals (<30 µm in the longest dimension) of insulin, thaumatin and HEWL, in their respective crystallization buffers, were mixed with each cryoprotectant and loaded onto a chip. The chips were plunge-frozen in liquid nitrogen and a 60° wedge of diffraction data was collected from ten crystals per chip. After the data had been processed, two metrics were used to assess crystal quality: the highest resolution (at CC_1/2_ = 0.3) and the mosaicity. The mosaicity was observed as an indicator of slight changes to the crystal structure that might occur without the loss of diffraction resolution. Given the blotting during chip loading, it is possible that the crystals may begin to start drying. Changes in mosaicity may capture this.

**Figure 7:**
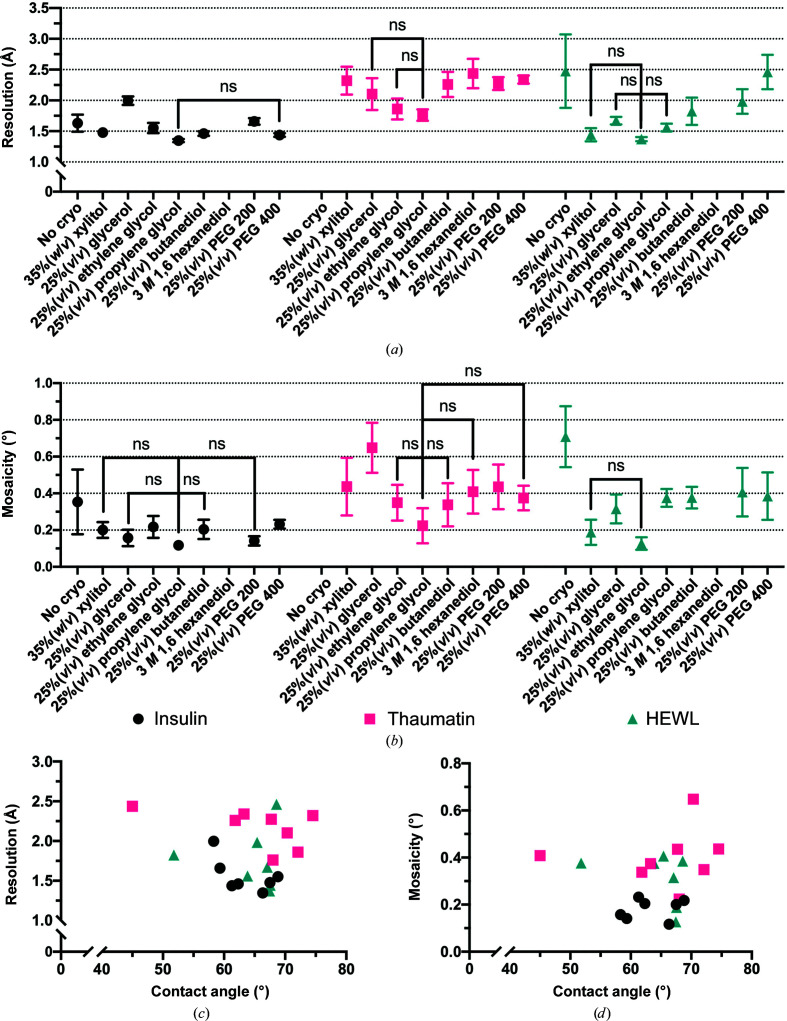
Comparing the resolution (*a*) and mosaicity (*b*) of insulin, thaumatin and HEWL crystals mounted on chips with different cryoprotectants. The high-resolution limit was set at the point where CC_1/2_ = 0.3. The mean and 95% confidence limits of each measurement have been plotted. Data points that are not significantly different (ns), as determined by a one-way ANOVA comparison, from the highest resolution or lowest mosaicity point for each protein have been marked. (*c* *, d*) The contact angle of each cryoprotectant for each protein/buffer plotted against their resolution and mosaicity, respectively. A Pearson correlation analysis was performed for the cryoprotectants in each protein/buffer and none were found to be significantly correlated.

For the insulin and thaumatin crystals, 25%(*v*/*v*) propylene glycol produced both the highest resolution diffraction (1.35 ± 0.03 and 1.76 ± 0.09 Å, respectively) and also the smallest mosaicity (0.12 ± 0.02° and 0.22 ± 0.10°, respectively). For HEWL, 25%(*v*/*v*) ethylene glycol produced the highest resolution (1.37 ± 0.03) and the lowest mosaicity (1.13 ± 0.03°). However, it should be noted that none of these values were significantly different from all of the other data points (Fig. 7 ▸ *a* and 7 ▸ *b*).

These analyses indicated that the link between the wetting properties of crystal loading buffers, quantified by the contact angle, and the resulting data quality, as determined by the resolution at CC_1/2_ = 0.3 and the mosaicity, was weak. This observation was confirmed by performing a Pearson correlation analysis (Figs. 7 ▸ *a* and 7 ▸ *b*) between the contact-angle measurement and the data-collection metrics. For this series of cryoprotectants, protein crystals and buffers, the chip–solution contact angle was not statistically correlated to either the resolution or the mosaicity from the diffraction data. This implies that the wetting of the chip surface also does not clearly correlate to the diffraction data quality. The protective effect of cryoprotectants in the investigated cases of HEWL, insulin and thaumatin therefore does not rely solely on chip wetting properties as initially hypothesized.

### 3.5. Comparison of crystals loaded into loops, cryo-meshes and chips   

**Figure 8:**
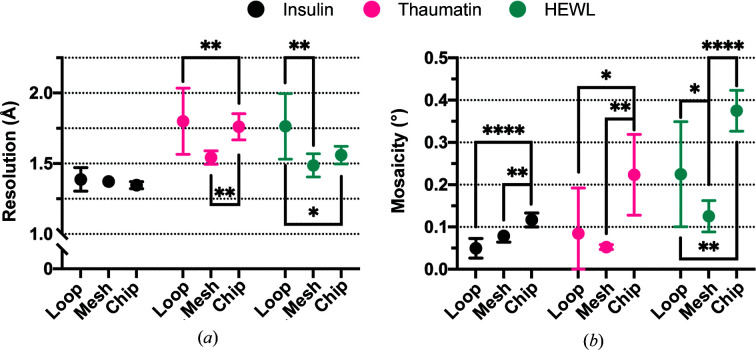
A comparison of the mean resolution (*a*) and mosaicity (*b*) of diffraction data collected from crystals mounted in loops, meshes or chips. The high-resolution limit was set at the point where CC_1/2_ = 0.3. The mean and 95% confidence limits of each measurement have been plotted. The asterisks indicate the significance of the *p*-value obtained from a one-way ANOVA comparison between the different mount (*, <0.0332; **, <0.0021; ***, <0.0002; ****, <0.0001).

The quality of the diffraction data collected from crystals loaded onto chips was compared with those obtained using the standard crystal mounts, loops and meshes, which have a considerably smaller area and therefore a much lower loading capacity than the chips. The same insulin, thaumatin and HEWL crystals were loaded into the different mounts, and diffraction data were collected and processed. 25% propylene glycol in precipitant was used as the cryoprotectant in each case. Data were collected from ten crystals from each mount, and their mean resolution and mosaicity were compared using a one-way ANOVA comparison. The results are shown in Fig. 8 ▸.

The very best resolution and lowest mosaicity over all three microcrystal samples were generally observed from the mesh-loaded samples. Similarly high resolutions were obtained from the chips, albeit with a trend to poorer mosaicity, within an acceptable range. The chips generally yielded better resolutions than the loops, although again with a slightly poorer mosaicity. We conclude that loading crystals onto the chips provides sufficiently similar levels of crystal preservation as standard mounts up to a high resolution range, allowing the low background and high loading area of the chips in serial crystallography experiments to be made use of.

## 4. Discussion   

We have created a novel crystal-mounting support (Karpik *et al.*, 2020 ▸) and demonstrated its utility in a variety of common and more challenging cases. Our approach to sample deposition was to immobilize crystals on the chip surface and leave only a thin liquid meniscus around each crystal to minimize the background scattering in the experimentally relevant resolution range. It was experimentally determined that the addition of cryoprotectant to the crystallization buffer was essential for maintaining the crystal integrity during cryocooling (Figs. 7 ▸ *a* and 7 ▸ *b*). The addition of cryoprotectant also appeared to improve the loading process and to help to prevent the crystals from drying (Fig. 3 ▸).

In an attempt to identify general cryoprotectant properties that would aid future users of the chips in their choice of cryoprotectant, we investigated how the solution–surface contact angle, the principal parameter for determining the interaction between a surface and a liquid, relates to diffraction resolution, a common metric of crystal quality. Unfortunately, it is not clear what properties an ideal cryoprotectant should have to facilitate chip loading. This said, it is already not clear *a priori* which cryoprotectant should be used for a new protein crystal and the process is likely to require some optimization (Garman & Owen, 2006 ▸).

Based on our experience with the proteins in this study, we propose the following steps and starting conditions for optimization of the sample deposition. First of all, depositing the native sample directly onto the chips should be attempted in order to evaluate the need for optimization. In some cases, such as EcR2a and the membrane-protein complex rhodopsin–miniG_o_, direct deposition provided satisfactory results. The mother liquor might already contain either cryoprotectants or components that induce a favorable spreading of the crystals across the chip (propylene glycol, short- or medium-chain PEGs or surfactants). If the native deposition is not satisfactory, a range of cryoprotectant solutions can then be assayed. Based on the limited study performed here, a range of cryoprotectants will be suitable for the various crystallization buffer types (mainly PEG, mainly salt or PEG and salt), with propylene and ethylene glycol being good options with which to start the optimization process. In the case of TD1, the ‘transfer solution’ was a composition of the precipitant in which the PEG 3350 was exchanged for PEG 400. When a surfactant is present, *i.e.* for lipid- or detergent-containing crystallization buffers, the wetting on the polymer film is usually sufficient. In this respect, the chips presented here represent an attractive alternative delivery method for sponge-phase-grown crystals. Supplementary Table S1 gives some suggestions for troubleshooting the initial steps of deposition optimization for new cases.

The data collected from proteins loaded onto the chips compares favorably with similar structures in the Protein Data Bank (PDB). It was interesting to contrast the data collected by small rotations with the stills data. Although a detailed comparison between these two data-collection modes was limited by the different data-processing software, doses per position (ten times higher for the wedges) *etc.*, the final resolutions were similar. This suggests that the intrinsic diffraction limit of the samples using this preparation method may have been reached with both data-collection methods.

The resolution of the rhodopsin–miniG_o_ complex on the chip was modest (Table 2 ▸), but was comparable to the best resolution from crystals harvested in a conventional manner from the same drops as a control (Supplementary Table S2 and Fig. S7). The 4 Å resolution obtained here does not reach the best reported resolution of 3.1 Å, which was obtained using five different crystal data sets from among the hundreds screened in Tsai *et al.* (2018 ▸). However, the resolution determined here is still an achievement for a membrane-protein complex, and is certainly representative of a step on the path to optimization. Blotting of the sponge phase was successful on the silanized chips; as shown by investigation of the background signal (Supplementary Fig. S8*b* ). This allowed a membrane-protein data set to be collected from the visible crystals (Supplementary Fig. S8*a* ). However, the procedure for sponge-phase blotting still requires substantial improvements and is not yet suitable for crystals that exist in both the cubic and sponge phase, which is a common occurrence in LCP crystallization trials.

Our results illustrate how polymer chips can be used to facilitate harvesting of surfactant-containing drops by direct deposition of the drops onto chips with a micropipette. This allows insight to be gained into the diffraction quality of many crystals randomly deposited on the chips rather than that of an arbitrary selection of cumbersomely harvested crystals. This is particularly valuable at the crystal-optimization stage.

The chips were designed to create a crystal support which limits additional background signal to an absolute minimum. The ultimate success of this can be seen in Fig. 6 ▸, where crystals loaded onto a chip would only incur three and one extra detector counts at 3.1 and 2.1 Å resolution, respectively. The impact on background scatter is also greatly reduced when compared with micro-mesh supports. However, the removal of this solution does come with a potential cost: crystal drying.

The comparison of data quality between the same crystals loaded into loops, micro-meshes and chips provided remarkable findings. Although the final resolution from each mount was relatively comparable, the mosaicity of the crystals on each was not. The chip-immobilized crystals were consistently, and significantly, more mosaic than their counterparts loaded in either loops or meshes. It is probable that, given the blotting process during chip-loading, this increase in mosaicity is due to a decrease in crystal hydration. This said, there does not appear to be a loss of structure quality, but it is of note as the difference between a ‘well loaded chip’ and a ‘poorly loaded chip’ may be minimal. Ultimately, the choice of mount should be dictated by the experiment being performed. Here, the advantage of the chips is their size and therefore the number of crystals that can be loaded on a single mount.

Finally, an advantage of the thin polymer film used for chip fabrication is its transparency to light, which means that loaded crystals can be easily seen. This visibility can be used for offline or online optical prelocation, *i.e.* to identify and center the crystals directly without the need for diffraction-based rastering to find crystals. Figs. 2 ▸(*a*)–2 ▸(*c*) show examples of images offering ideal conditions for offline optical pre­location, with homogeneous illumination and small pixel size for easier automatic detection of small features on the images (Supplementary Fig. S9). Software such as *ImageJ* (Schneider *et al.*, 2012 ▸) can be used to prelocate crystals without the use of X-rays.

## 5. Conclusion and outlook   

We have demonstrated the use of large-area, perforated thin polymer membranes for low-background crystallographic serial data collection at a microfocus synchrotron beamline. Our loading method relies on blotting away residue deposition solution such that a thin film is left only around the crystals, thus preserving the crystals while offering low-background conditions. We tested this approach on a variety of cases and derived a series of guidelines for other proteins from these experiments. Although our investigations focused on cryogenic conditions, the goal is to also create a room-temperature chip. Preliminary tests suggest that the chips can be used for room-temperature data collection by replacing the cryojet with a humid stream or by enclosing the loaded chip to preserve the crystal humidity. We are also investigating ways to implement additional micro-topography to the chip surface, so that the chips are more applicable for low-symmetry space groups prone to preferential orientations.

## Supplementary Material

PDB reference: ribonucleotide reductase radical-generating subunit, 7ai8

PDB reference: 7ai9

PDB reference: hen egg-white lysozyme, 7ac2

PDB reference: thaumatin, 7ac3

PDB reference: insulin, 7ac4

PDB reference: tubulin–DARPin D1 complex, 7ac5

PDB reference: sponge-phase-grown PepTst2, 7ac6

Supplementary Figures and Tables. DOI: 10.1107/S2059798321007324/wa5131sup1.pdf

## Figures and Tables

**Table 1:** Crystal dimensions and deposition solutions

Protein	Crystal size (µm)	Deposition solution
Lysozyme	15 × 10 × 10	30% propylene glycol, 70% precipitant
Thaumatin	10 × 5 × 5 to 20 × 10 × 10	30% propylene glycol, 70% precipitant
Insulin	20 × 20 × 20	30% propylene glycol, 70% precipitant
TD1-rot	100 × 10 × 5 to 1000 × 100 × 50	Solution analogous to the precipitant but replacing PEG 3000 with PEG 400
TD1-still	100 × 10 × 5 to 1000 × 100 × 50	Solution analogous to the precipitant but replacing PEG 3000 with PEG 400
EcR2a-rot	150 × 50 × 50	Precipitant
EcR2a-still	150 × 50 × 50	Precipitant
Rhodopsin–miniG_o_	100 × 10 × 10 to 200 × 15 × 15	None (directly from the drop)
PepT_St_	30 × 20 × 20	None (directly from the syringe)

**Table 2:** Data-collection and refinement statistics for the various cases studied Values in parentheses are for the highest resolution shell. All data were collected from a single chip on the SLS beamlines at 12.4 keV.

Protein	Lysozyme	Thaumatin	Insulin	TD1-rot	TD1-still	EcR2a-rot	EcR2a-still	Rhodopsin–miniG_o_	PepT_St_
Data-collection parameters									
Beamline	PXI	PXI	PXII	PXI	PXI	PXI	PXI	PXI	PXII
Data-collection mode	Oscillations	Oscillations	Oscillations	Oscillations	Stills	Oscillations	Stills	Oscillations	Oscillations
Beam size (µm)	5 × 7	10 × 10	18 × 10	10 × 10	10 × 10	10 × 10	10 × 10	20 × 10	18 × 10
Flux (photons s^−1^)	2 × 10^11^	2 × 10^11^	4 × 10^11^	6 × 10^11^	6 × 10^11^	6 × 10^11^	6 × 10^11^	1.2 × 10^12^	4 × 10^11^
Oscillation (°)	0.2	0.2	0.2	0.2	Stills	0.2	Stills	0.2	0.2
Exposure (s)	0.1	0.1	0.1	0.1	0.5	0.1	0.5	0.1	0.1
Total oscillation (°)	10	5	10	10		10		10	10
Dose per crystal (MGy)	0.52	0.81	1.8	2.2	0.23	1.5	0.14	1.9	1.4
Collection positions	82	70	30	120	33568	60	54000	116	68
Indexing rate (%)	93	70	90	82	21	100	15	99	24
Merged wedges/stills	71	42	24	81	7118	47	7879	89	15
Unit-cell parameters									
Space group	*P*4_3_2_1_2	*P*4_1_2_1_2	*I*2_1_3	*P*2_1_	*P*2_1_	*P*6_1_22	*P*6_1_22	*P*6_1_	*C*222_1_
*a*, *b*, *c* (Å)	79.02, 79.02, 37.08	58.00, 58.00, 150.31	77.96, 77.96, 77.96	73.32, 92.03, 83.54	73.51, 90.92, 82.49	90.51, 90.51, 207.87	90.35, 90.35, 208.55	151.18, 151.18, 96.36	101.76, 110.23, 109.82
α, β, γ (°)	90.0, 90.0, 90.0	90.0, 90.0, 90.0	90.0, 90.0, 90.0	90.0, 96.8, 90.0	90.0, 97.9, 90.0	90.0, 90.0, 120.0	90.0, 90.0, 120.0	90.0, 90.0, 120.0	90.0, 90.0, 90.0
Data reduction									
Resolution (Å)	39.51–1.50 (1.54–1.50)	45.92–1.65 (1.69–1.65)	38.98–1.46 (1.50–1.46)	49.21–2.26 (2.32–2.26)	58.49–2.26 (2.30–2.26)	45.26–2.00 (2.05–2.00)	52.20–2.10 (2.13–2.10)	49.49–4.00 (4.10–4.00)	49.26–3.00 (3.08–3.00)
*R*_meas_	0.190 (7.196)	0.321 (3.931)	0.148 (3.22)	0.436 (8.368)	—	0.197 (11.05)	—	0.215 (53.09)	0.321 (1.506)
*R*_p.i.m._	0.036 (1.451)	0.114 (1.388)	0.055 (1.25)	0.152 (2.944)	—	0.034 (2.299)	—	0.069 (8.67)	0.184 (0.887)
*R*_split_	—	—	—	—	0.250 (1.209)	—	0.054 (0.748)	—	—
〈*I*/σ(*I*)〉	10.44 (0.44)	4.19 (0.54)	8.26 (0.70)	4.28 (0.64)	3.32 (1.04)	10.05 (0.48)	10.75 (1.74)	9.67 (0.38)	3.84 (0.78)
Completeness (%)	99.7 (100)	99.5 (100)	99.9 (99.7)	94.1 (94.8)	100 (99.46)	100 (99.9)	100 (96.7)	99.7 (97.1)	94.7 (92.0)
Multiplicity	26.5 (24.6)	7.98 (8.02)	7.16 (6.64)	8.22 (8.08)	88.5 (57.4)	26.4 (23.1)	201 (15.2)	47.1 (37.5)	3.05 (2.88)
CC_1/2_	0.997 (0.172)	0.986 (0.144)	0.997 (0.188)	0.974 (0.126)	0.948 (0.189)	0.999 (0.183)	0.997 (0.383)	0.999 (0.274)	0.974 (0.217)
Wilson *B* factor (Å^2^)	26.59	22.72	19.65	40.9	35.70	59.93	41.87	184	57.77
No. of reflections	19098 (1880)	31813 (3127)	13869 (1379)	49614 (4049)		34438 (2439)	30094 (2463)		12579 (1192)
Refinement									
*R*_work_/*R*_free_	0.179/0.204	0.166/0.210	0.173/0.177	0.216/0.255		0.170/0.191	0.190/0.219		0.240/0.290
No. of atoms									
Total	1129	1889	484	8018		2944	2897		3976
Protein	998	1576	426	7808		2792	2792		3516
Ligand/ions	6	22	6	69		2	2		441
Water	125	291	52	141		150	103		19
*B* factors (Å^2^)									
Overall	31.46	26.24	30.01	56.85		78.96	57.43		67.33
Protein	30.38	23.72	27.68	57.08		79.09	57.69		66.33
Ligand/ions	35.54	39.79	75.72	49.99		58.59	40.35		76.31
Water	39.84	38.84	43.87	47.82		76.85	50.83		43.14
R.m.s. deviations									
Bond lengths (Å)	0.009	0.017	0.01	0.006		0.009	0.007		0.002
Bond angles (°)	1.05	1.46	1	0.81		0.98	0.83		0.5
Ramachandran plot									
Favored (%)	98.43	98.53	100	98.53		99.7	98.81		98.43
Allowed (%)	1.57	1.47	0	1.18		0.3	1.19		1.57
Outliers (%)	0	0	0	0.29		0	0		0
Rotamer outliers (%)	0	0.58	0	0.98		1.62	1.3		2.2
Clashscore	3.59	4.17	3.54	5.6		2.71	2.89		7.3
PDB code	7ac2	7ac3	7ac4	7ac5		7ai9	7ai8		7ac6

**Table 3:** A list of the different cryoprotectants and crystallization buffer components used to test the relationship between the ‘wettability’ of a crystallization solution and the quality of the diffraction data The buffer component concentrations are all given as final concentrations, *i.e.* after the protein and crystallization buffers and cryoprotectant have all been mixed.

Cryoprotectants	Insulin buffer (mainly PEG)	Thaumatin buffer (mainly salt)	HEWL buffer (PEG and salt)
35%(*w*/*v*) xylitol	25 m*M* Na_2_PO_4_ pH 10.5	20%(*w*/*v*) sodium potassium tartrate	10.5%(*w*/*v*) NaCl
25%(*v*/*v*) glycerol	5 m*M* EDTA	0.05 *M* bis-Tris propane pH 6.5	4%(*w*/*v*) PEG 6000
25%(*v*/*v*) ethylene glycol	12.5%(*w*/*v*) PEG 6000		0.1 *M* sodium acetate pH 3.0
25%(*v*/*v*) propylene glycol	0.05 *M* bis-Tris propane pH 7.5		
25%(*v*/*v*) butanediol	0.1 *M* NaBr		
3 *M* 1,6-hexanediol			
25%(*v*/*v*) PEG 200			
25%(*v*/*v*) PEG 400			
