# Supplementary material for: Versatile microporous polymer-based supports for serial macromolecular crystallography
Source: Acta Crystallogr D Struct Biol. 2021 Aug 23;77(Pt 9):1153–67. doi: 10.1107/S2059798321007324 (PMC8411977; doi:10.1107/S2059798321007324)
Supplement: Supplementary file 1 [file d-77-01153-sup1.pdf]

STRUCTURAL BIOLOGY

Volume 77 (2021)

Supporting information for article:

Versatile microporous polymer-based supports for serial macromolecular crystallography

Isabelle Martiel, John H. Beale, Agnieszka Karpik, Chia-Ying Huang, Laura Vera, Natacha Olieric, Maximilian Wranik, Ching-Ju Tsai, Jonas Mühle, Oskar Aurelius, Juliane John, Martin Högbom, Meitian Wang, May Marsh and Celestino Padeste

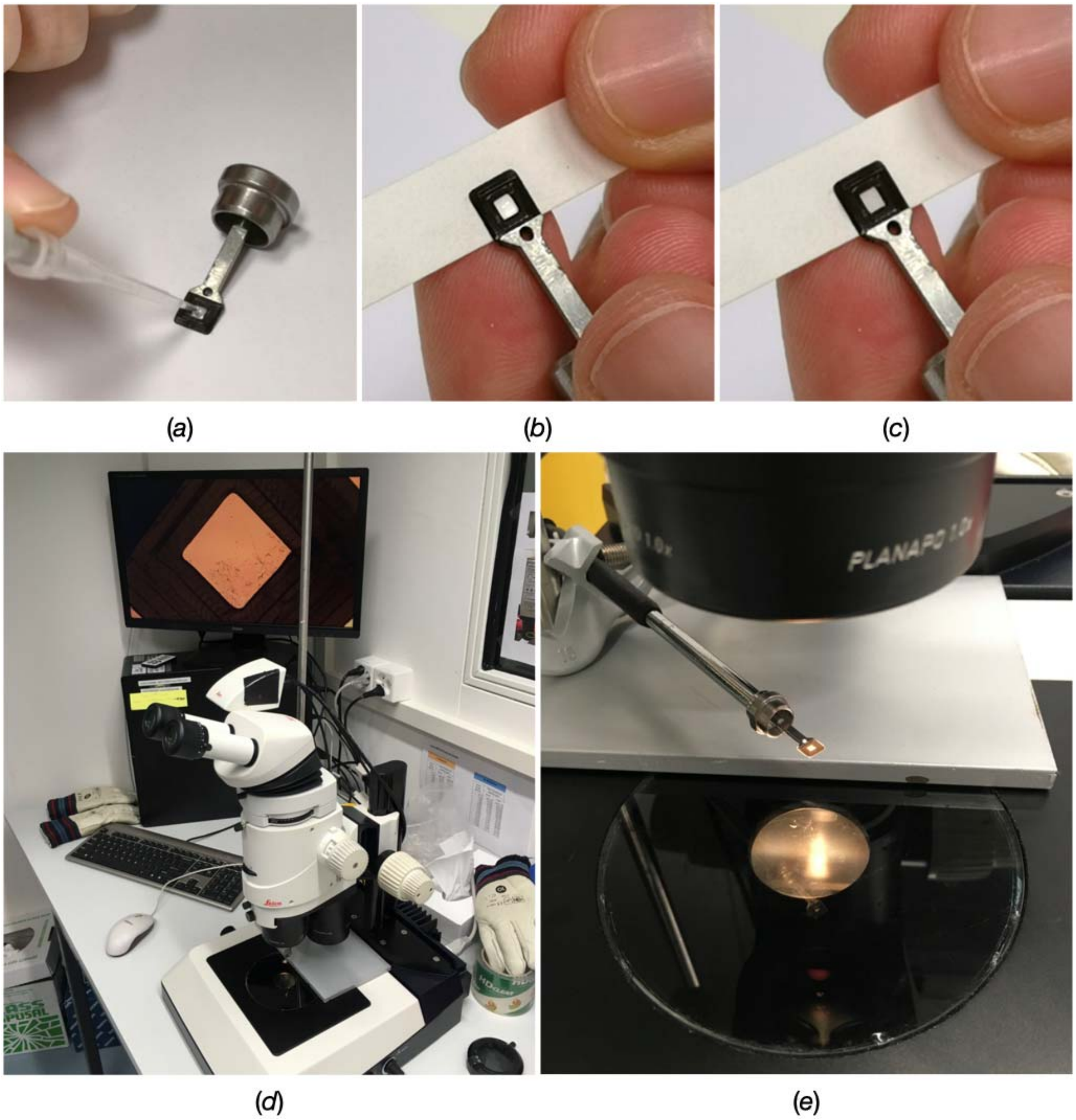

Figure S1 Deposition (*a*) and blotting (*b*, *c*) of the protein crystal suspension on the chips. While blotting, the droplet recedes through the chip [from (*b*) to (*c*)]. Leica MZ16 microscope with a chip mounted on a pin wand held in a retort stand (*d*, *e*). The setup used for blotting under a microscope.

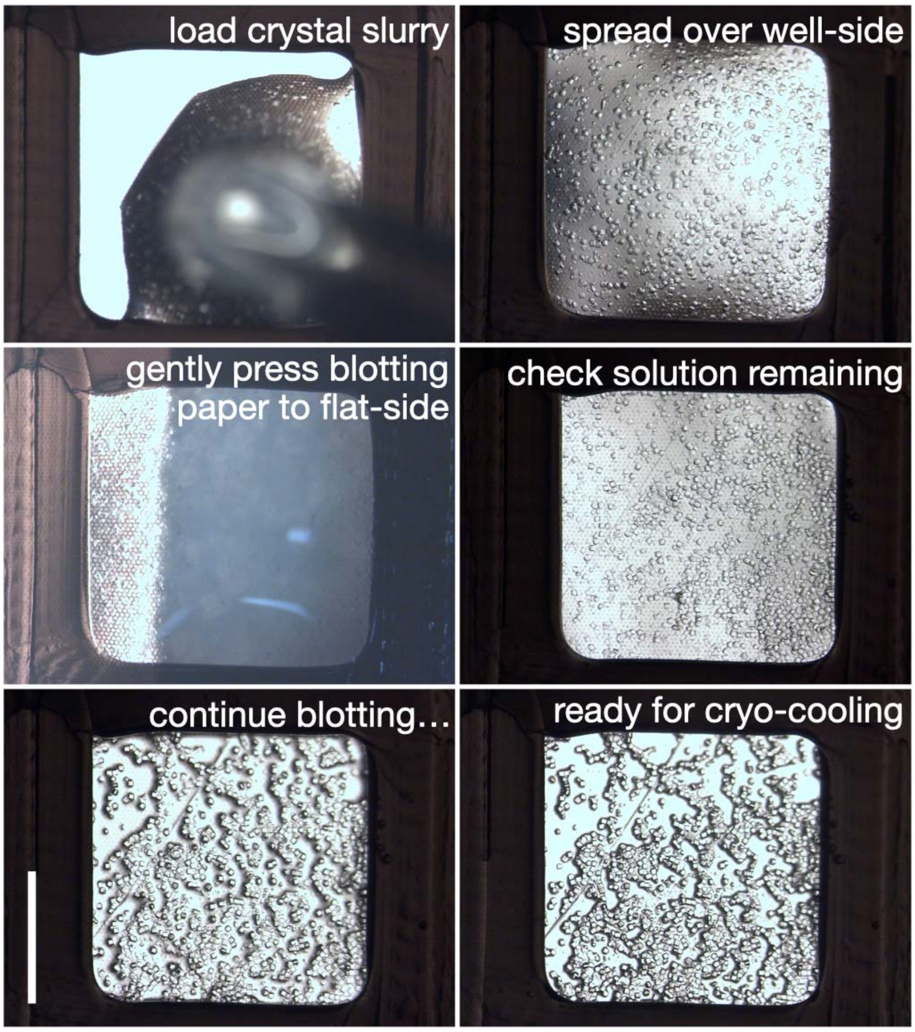

Figure S2 An example of blotting using insulin micro-crystals and the light microscope apparatus shown in Fig. S1. Sequence is from left to right and then from top to bottom. The scale bar in the bottom-left panel denotes 1 mm.

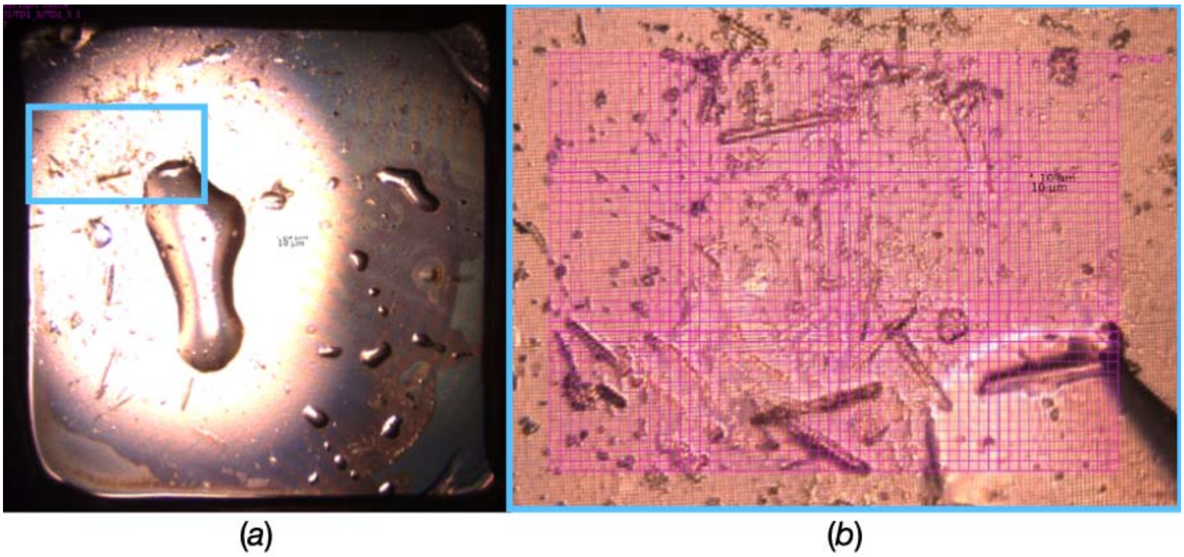

Figure S3 Non-ideal sample deposition on the chip surface, exemplified by the TD1 crystals. (*a*) An image of the complete chip surface. (*b*) A magnified image of the blue rectangle in (*a*). The crystals have either been deposited with too little solution remaining around them or with too much. To little solution will cause the crystals to dry, and too much will increase the background on the detector.

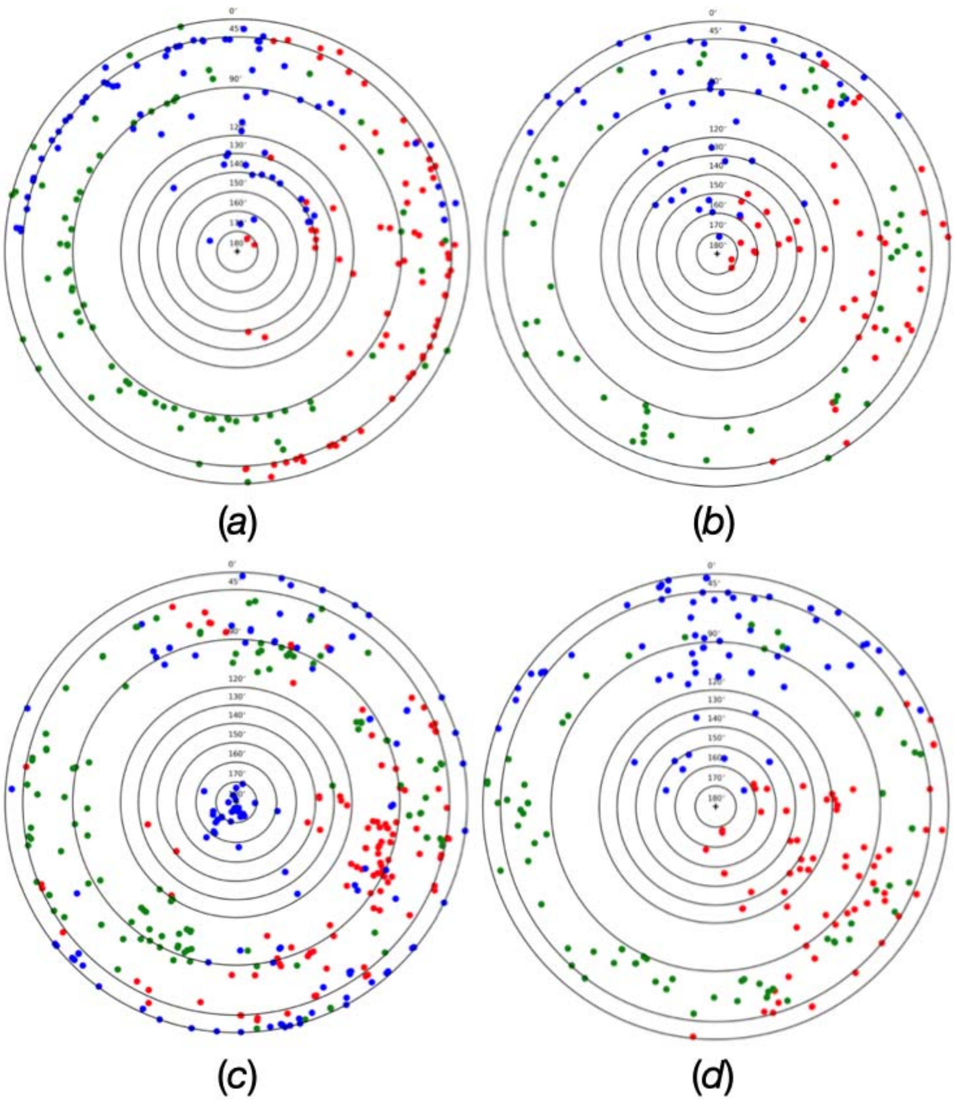

Figure S4 Lambert equal-area projection of crystal orientations of crystals on chips, extracted from the serial data processing pipeline output. (*a*) lysozyme microcrystals (*b*) thaumatin microcrystals, (*c*) TD1, long needles, space group P21 (*d*) EcR2a crystals. The direction of the *a* axis is shown in red, *b* in blue and *c* in green. Lysozyme microcrystals (*a*) tend to lie flat on the support, as shown by the *c* axis predominantly at 90 degrees angle. TD1 crystals (*c*) also lie flat, with many *b* axis at 0 or 180 degrees. The distribution is more homogeneous in the case of thaumatin (*b*) and Ecr2a (*d*).

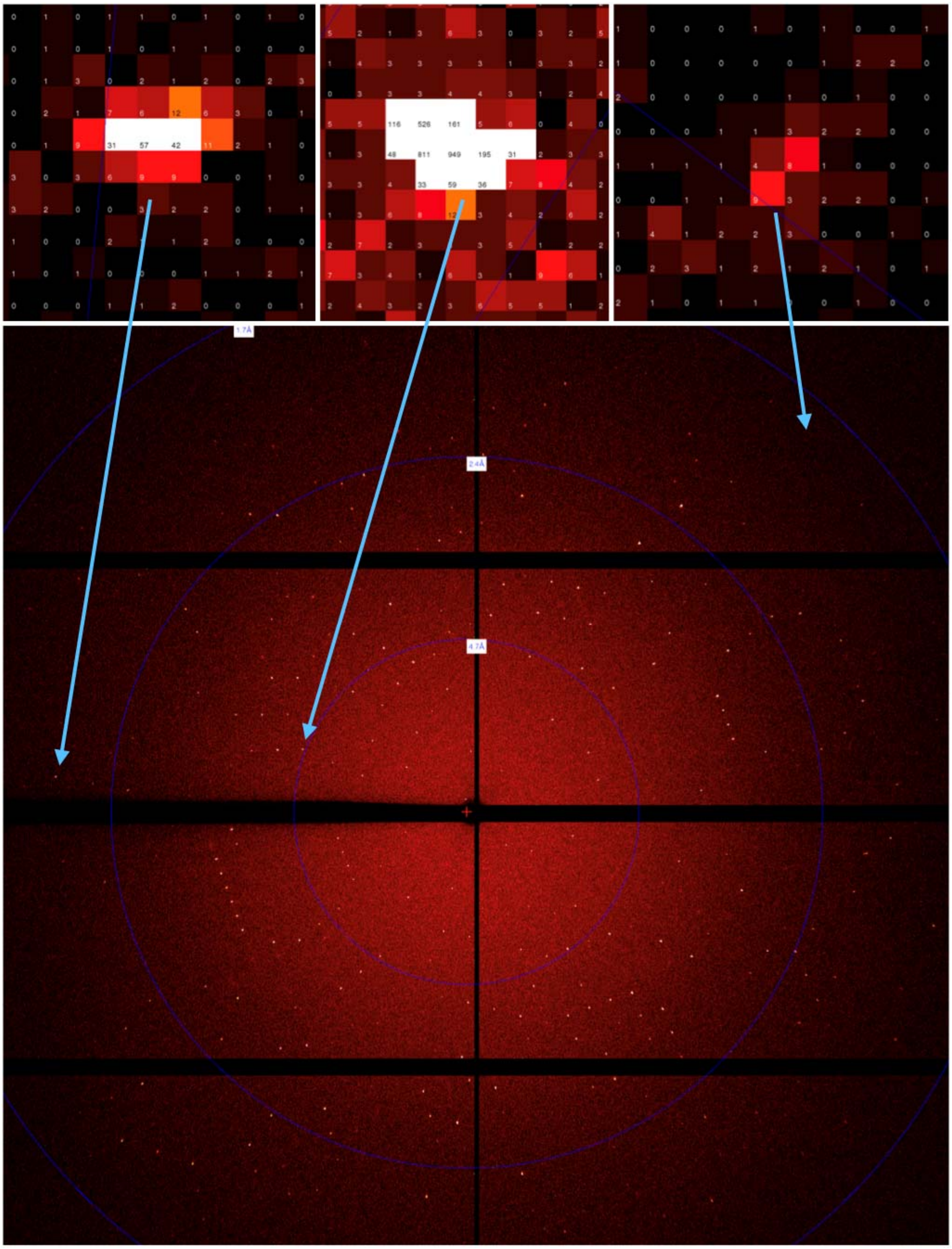

Figure S5 Representative diffraction pattern from a grid scan on a lysozyme crystal deposited on a chip (6e11 ph/s, 0.02 s exposure, Eiger 4M detector distance 130 mm, 12.4 keV).

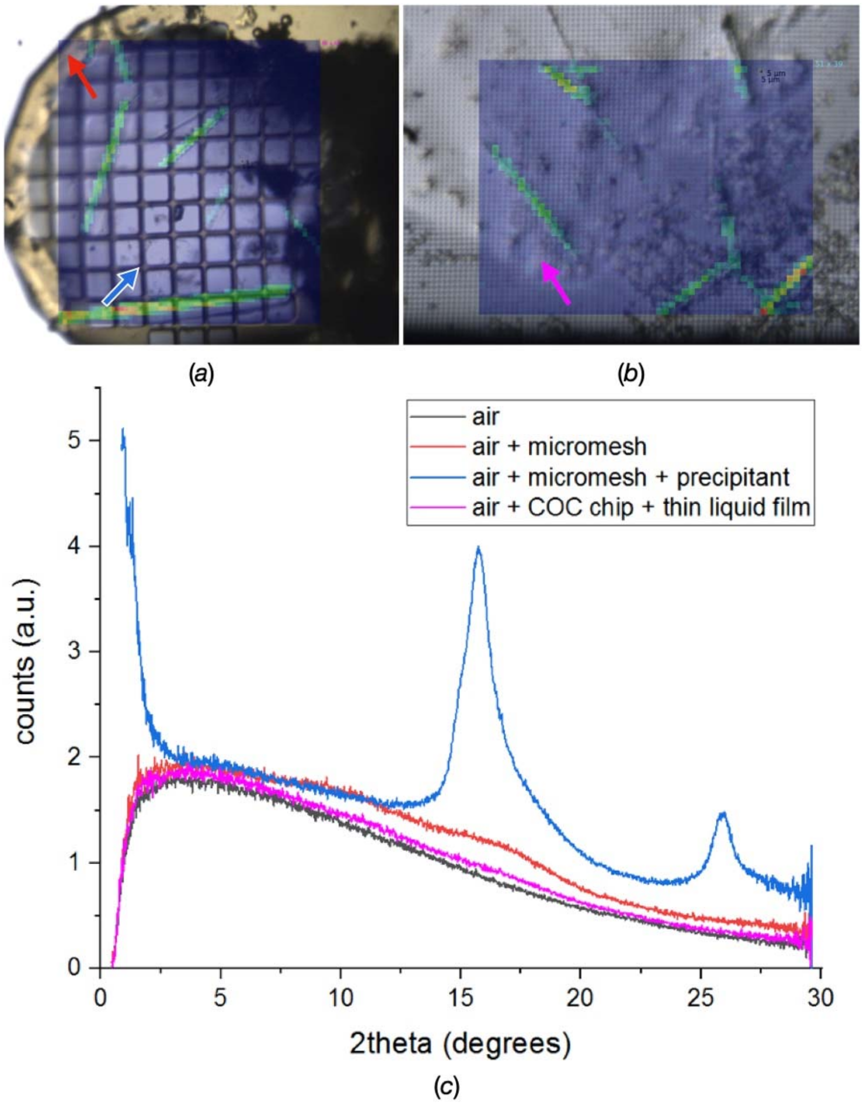

Figure S6 Background comparison between a COC chip and a standard polyimide micromesh measured on two TD1 samples. (*a*) and (*b*) Online microscope pictures from the TD1 crystals deposited on a polyimide micromesh (*a*) and on a COC chip (*b*). Arrows show the positions where the background curve of corresponding color was measured. (*c*) Measured background curves. The red curve shows the contribution from the plastic of the micromesh. The blue curve shows the contribution from both plastic and remaining liquid, with maxima at 3.4 Å and 2.1 Å resolution as expected for water-based media. As the thickness of liquid varies to a large extend, a representative curve with average signal height was chosen. This blue curve shows a much stronger background signal than the corresponding pink curve, showing the background signal obtained on a blotted TD1 sample deposited on a COC membrane chip. At the maxima, the background from the polyimide micromesh sample exceeds the contribution from the air scattering (black curve).

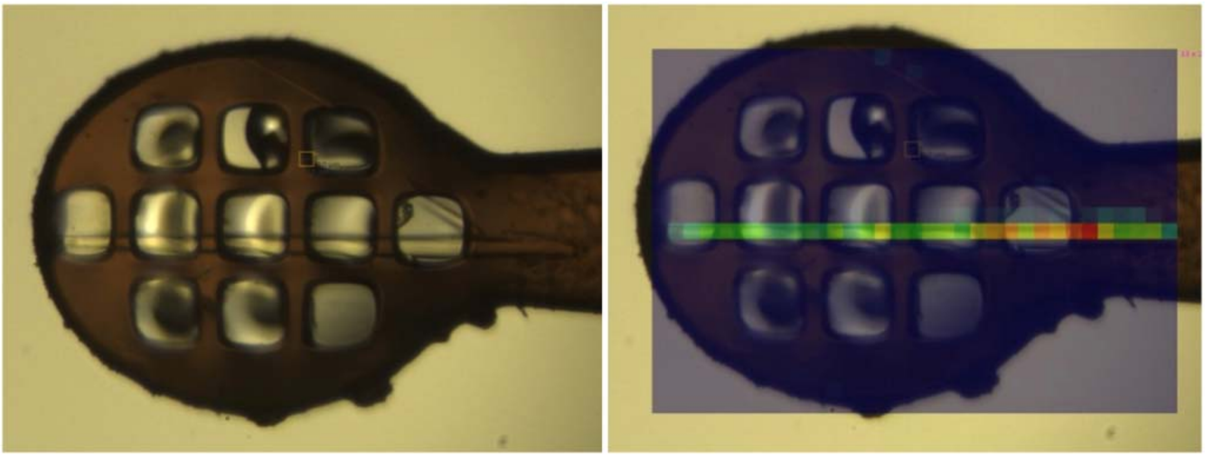

(a) (b)

Figure S7 Single crystal of rhodopsin-miniG$_o$, corresponding to the dataset which data collection statistics are shown in Table S1. This crystal was harvested in a conventional manner on polyimide micromeshes in the crystallization drops the chips were prepared from, before deposition on chip of the rest of the drop. On the right, the result of a raster scan to identify the best diffracting spot is shown as overlay.

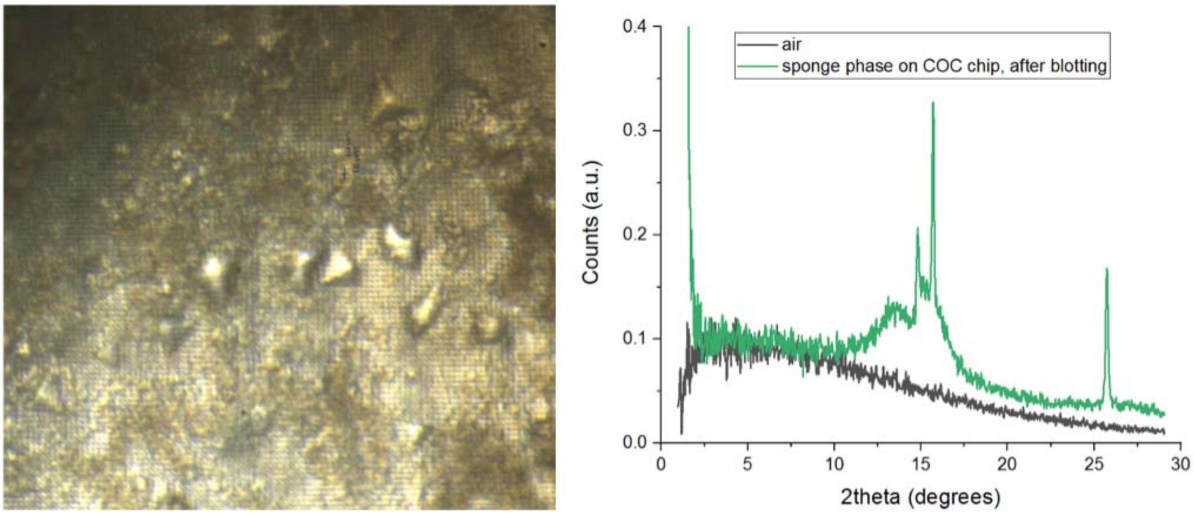

(a) (b)

Figure S8 Picture of blotted sponge phase (*a*) and corresponding background curve (*b*). The background signal from the residual sponge phase is comparable to that from air, indicating that a large part of the liquid phase was blotted away.

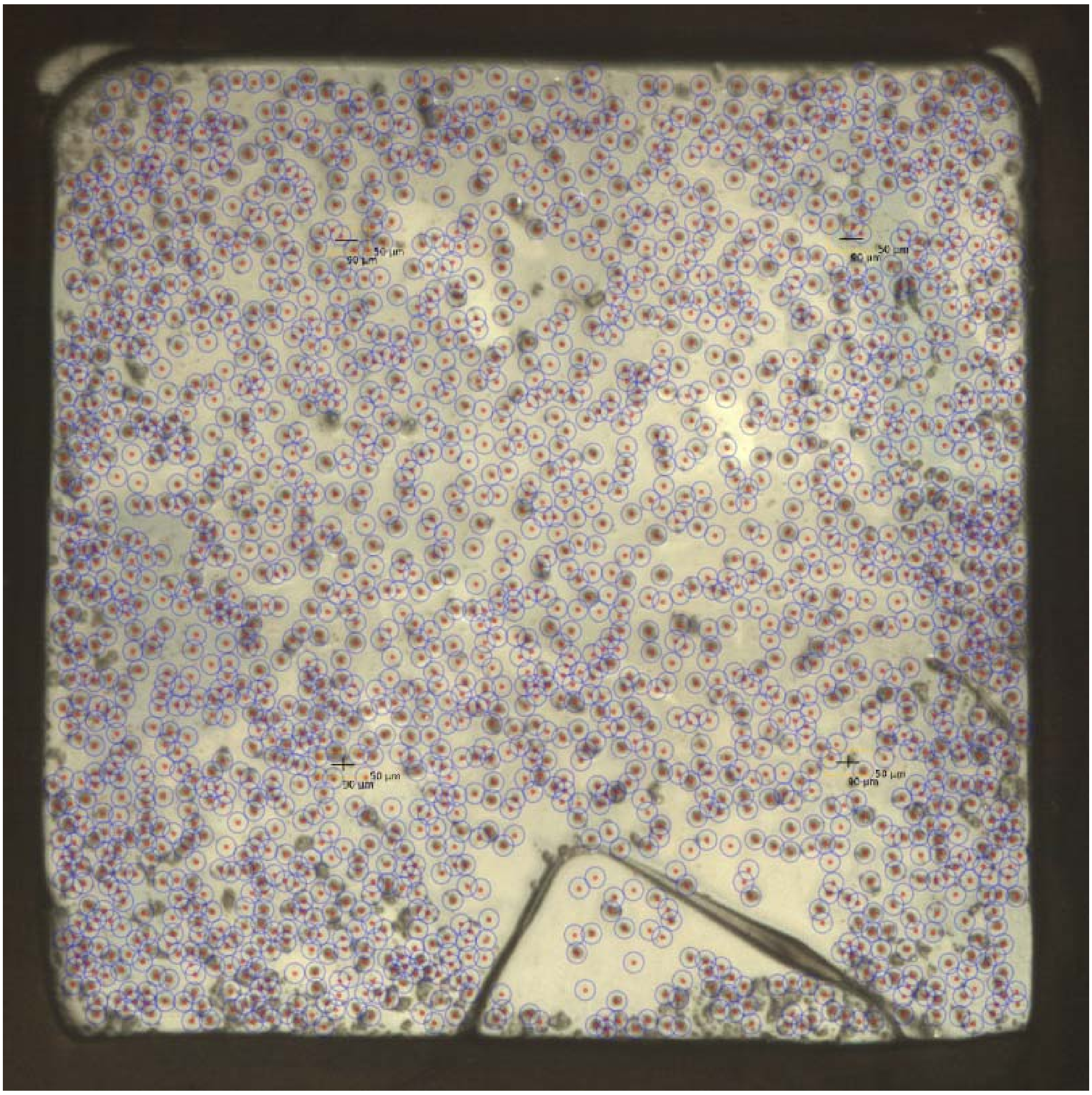

Figure S9 Example of semi-automatic detection and selection of 2125 crystals (red dots) on a stitched optical image of a chip carrying thaumatin microcrystals. The detection was done using the *Feature_Finder* function in *ImageJ*. Positions closer than 20 µm (radius of the blue circles) were excluded in the selection.

Table S1 Trouble-shooting guidelines for sample deposition on the COC membranes.

Issue	Possible indication
Ice rings or streaks on the diffraction patterns, icy or brownish areas visible on the chips	Cryoprotection is insufficient, possibly due to:<br>Too thick liquid layer left on the chip caused by insufficient blotting, blotting more may help<br>Too low concentration of cryoprotectant<br>Precipitate retaining too much liquid, increasing cryoprotectant amount may help
Protein crystals are dried out	Blotting too long<br>Waiting too long before freezing
Protein crystals don’t diffract as well as they should	Cryoprotection may be insufficient<br>The blotting may be inadequate<br>Crystals may have dried out<br>(frequent case) Crystals may not be compatible with the transfer solution, or stayed too long in there<br>Crystals may not like the fact of lying flat with a meniscus of liquid surrounding them, maybe try a thicker liquid layer with more cryoprotection
Big blobs of liquid at places	Optimize the transfer solution for a better wetting, blot a little longer
Background is high, diffuse rings	A precipitate may retain liquid on the chip<br>Blotting may be insufficient or did not happen at all

Table S2 Data collection statistics for a single crystal of rhodopsin-miniG$_o$. This crystal was the best found from screening a number of large crystals harvested in a conventional manner on polyimide micromeshes in the crystallization drops the chips were prepared from, before deposition on chip of the rest of the drop. Data collection parameters were: 360 degrees total range, detector distance 200 mm, 0.05 degrees and 0.02 s exposure per frame, 5% transmission of the full flux $5.6 \cdot 10^{11}$ ph/s, 10 µm x10 µm, 12.4 keV incoming X-ray energy.

Space group	P 61
Unit-cell parameters	
a, b, c (Å)	152.10, 152.10, 96.95
α, β, γ (°)	90.00, 90.00, 120.0
Resolution (Å)	19.23 - 4.30 (4.41 - 4.30)
Rmeas	0.65.6 (8.04)
<I/σ(I)> or signal-to-noise ratio	3.84 (0.94)
Completeness (%)	95.3 (94.9)
Multiplicity	21.9 (21.4)
CC1/2	0.992 (0.08)
